# Supplementary material for: Insertive condom-protected and condomless vaginal sex both have a profound impact on the penile immune correlates of HIV susceptibility
Source: PLoS Pathog. 2022 Jan 4;18(1):e1009948. doi: 10.1371/journal.ppat.1009948 (PMC8769335; doi:10.1371/journal.ppat.1009948)
Supplement: S1 Fig — Cytokine concentrations (pg/swab) at baseline from swabs of the coronal sulcus and penile shaft (N = 37). Red dotted line represents LLOD for immune parameter. Statistical comparisons were performed using two-tailed Mann-Witney U test. (DOCX) [file ppat.1009948.s001.docx]

S1 Fig. Baseline penile cytokine levels.

**
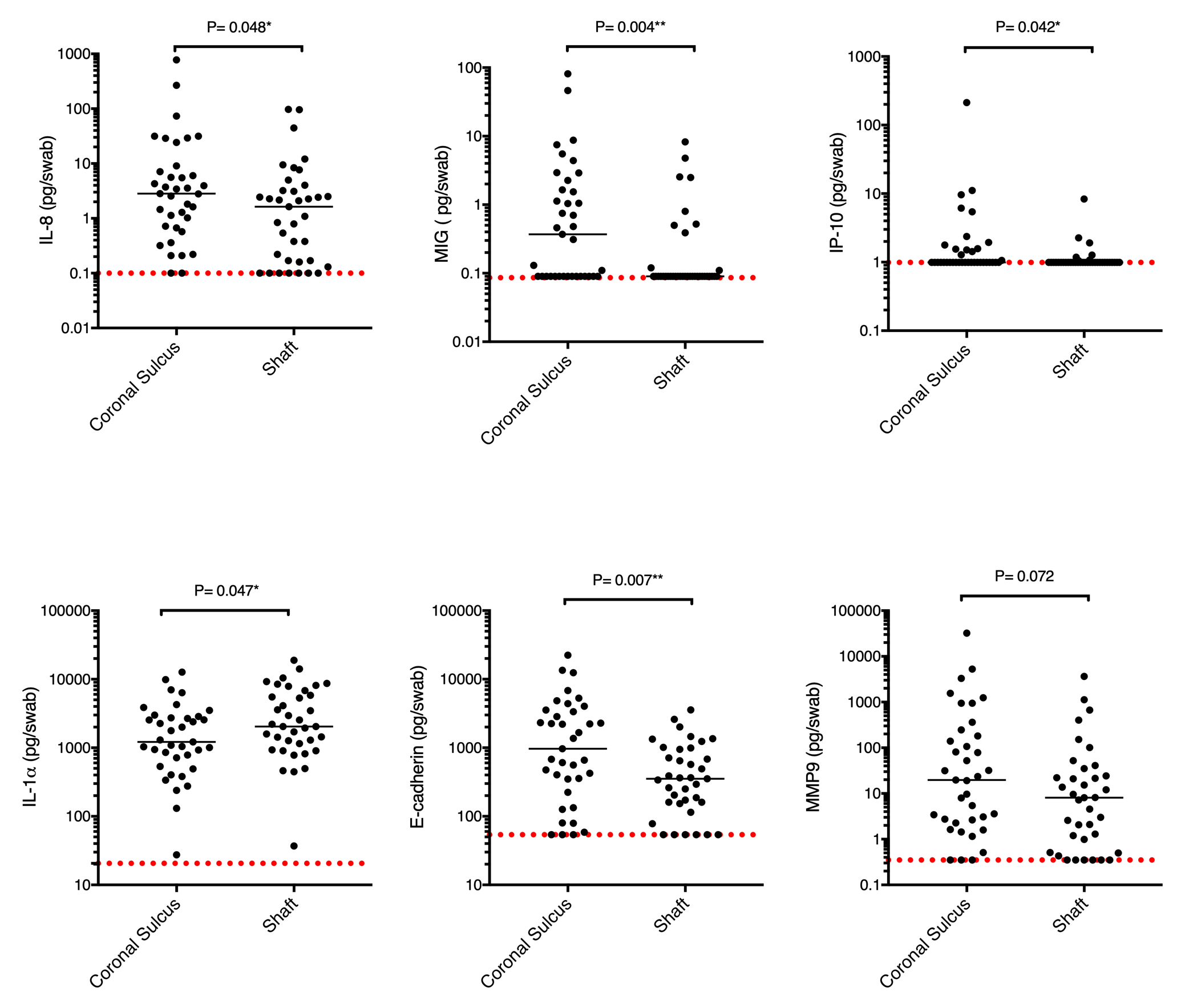
**

**S1 Fig. Baseline penile cytokine levels.**

Cytokine concentrations (pg/swab) at baseline from swabs of the coronal sulcus and penile shaft (N=37). Red dotted line represents LLOD for immune parameter. Statistical comparisons were performed using two-tailed Mann-Witney U test.
