# Supplementary material for: Insertive condom-protected and condomless vaginal sex both have a profound impact on the penile immune correlates of HIV susceptibility
Source: PLoS Pathog. 2022 Jan 4;18(1):e1009948. doi: 10.1371/journal.ppat.1009948 (PMC8769335; doi:10.1371/journal.ppat.1009948)
Supplement: S2 Fig — Unsupervised hierarchical clustering was used to visualize the fold change in penile cytokine concentrations 1 hour, 7 hours and 72 hours after sex, based on (A) condom use and (B) penile circumcision status. Scale denotes log2 transformed fold changes ranging from -12.15 to 15.54, and 1 represents no change. The numbers of participants with paired samples are: at 1hr n = 38; at 7hrs n = 38 and at 72hrs n = 34. (DOCX) [file ppat.1009948.s002.docx]

S2 Fig. Unsupervised hierarchical clustering of cerivco-vaginal cytokine changes after sex.

A)


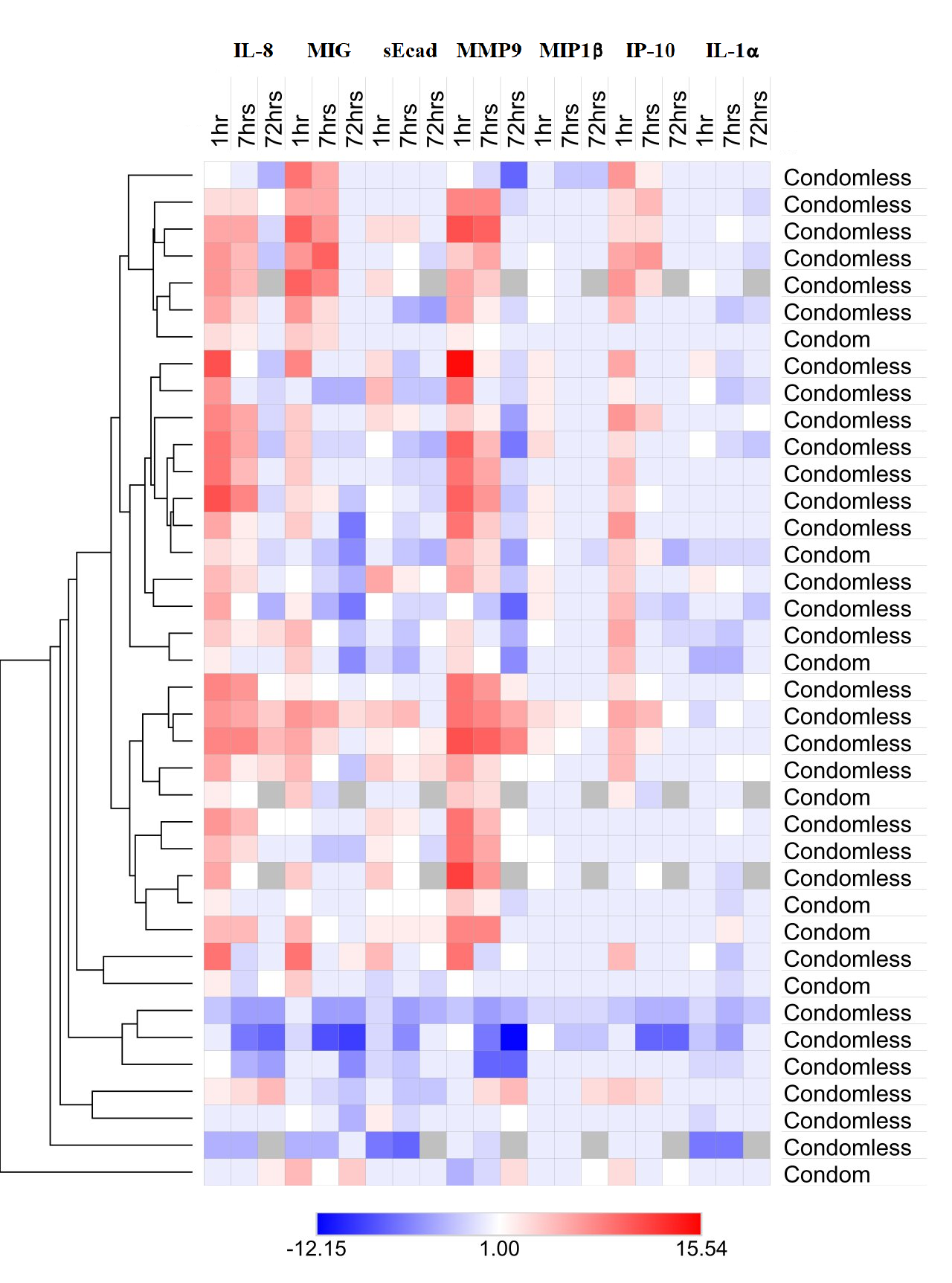


**B)**


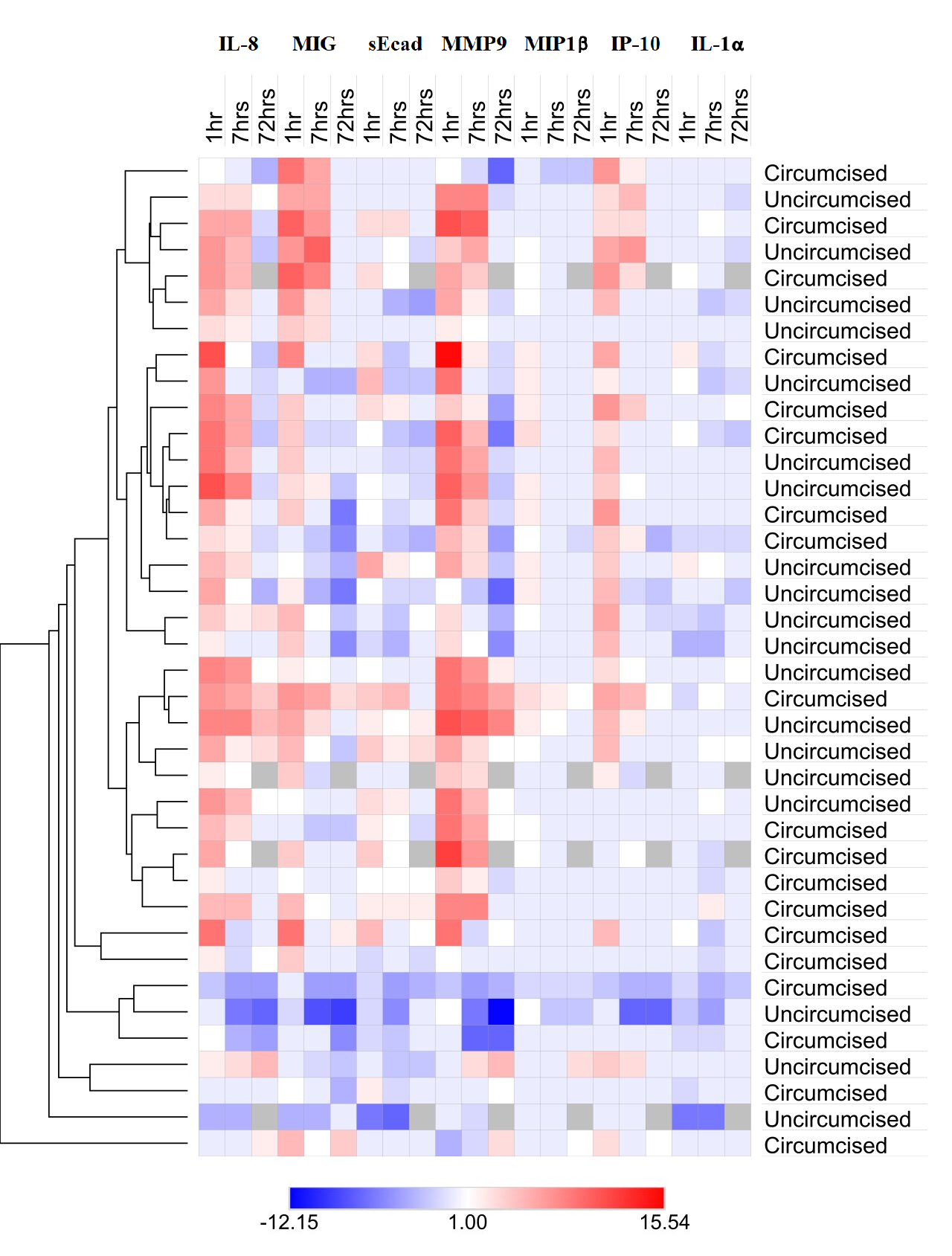


**S**2 **Fig. Unsupervised hierarchical clustering of penile cytokine changes after sex**. Unsupervised hierarchical clustering was used to visualize the fold change of cytokine concentrations 1 hour, 7 hours and 72 hours after sex based on (A) sex group (B) circumcision status. Scale denotes log2 transformed fold changes ranging from -12.15 to 15.54, and 1 is no change. The numbers of participants with paired samples are in 1hr=38 in 7hrs=38 and in 72hrs=34.
