# Supplementary material for: Insertive condom-protected and condomless vaginal sex both have a profound impact on the penile immune correlates of HIV susceptibility
Source: PLoS Pathog. 2022 Jan 4;18(1):e1009948. doi: 10.1371/journal.ppat.1009948 (PMC8769335; doi:10.1371/journal.ppat.1009948)
Supplement: S3 Fig — Time course of changes of (A) IL-1α (pg/swab) (B) IL-8 (pg/swab) (C) IP-10 (pg/swab) (D) MIG (pg/swab) (E) MIP-1β (pg/swab) (F) E-cadherin (pg/swab), and (G) MMP9 (pg/swab) after condomless sex (N = 30) and condom-protected sex (N = 8). The red dotted line represents immune parameter LLOD, and red numbers the median cytokine concentration at each visit. Statistical comparisons were performed using two-tailed Wilcoxon Signed Ranked test. (DOCX) [file ppat.1009948.s003.docx]

**S3 Fig. Impact of penile-vaginal sex on penile shaft immunology.**

**Condomless sex Condom-protected sex**

**
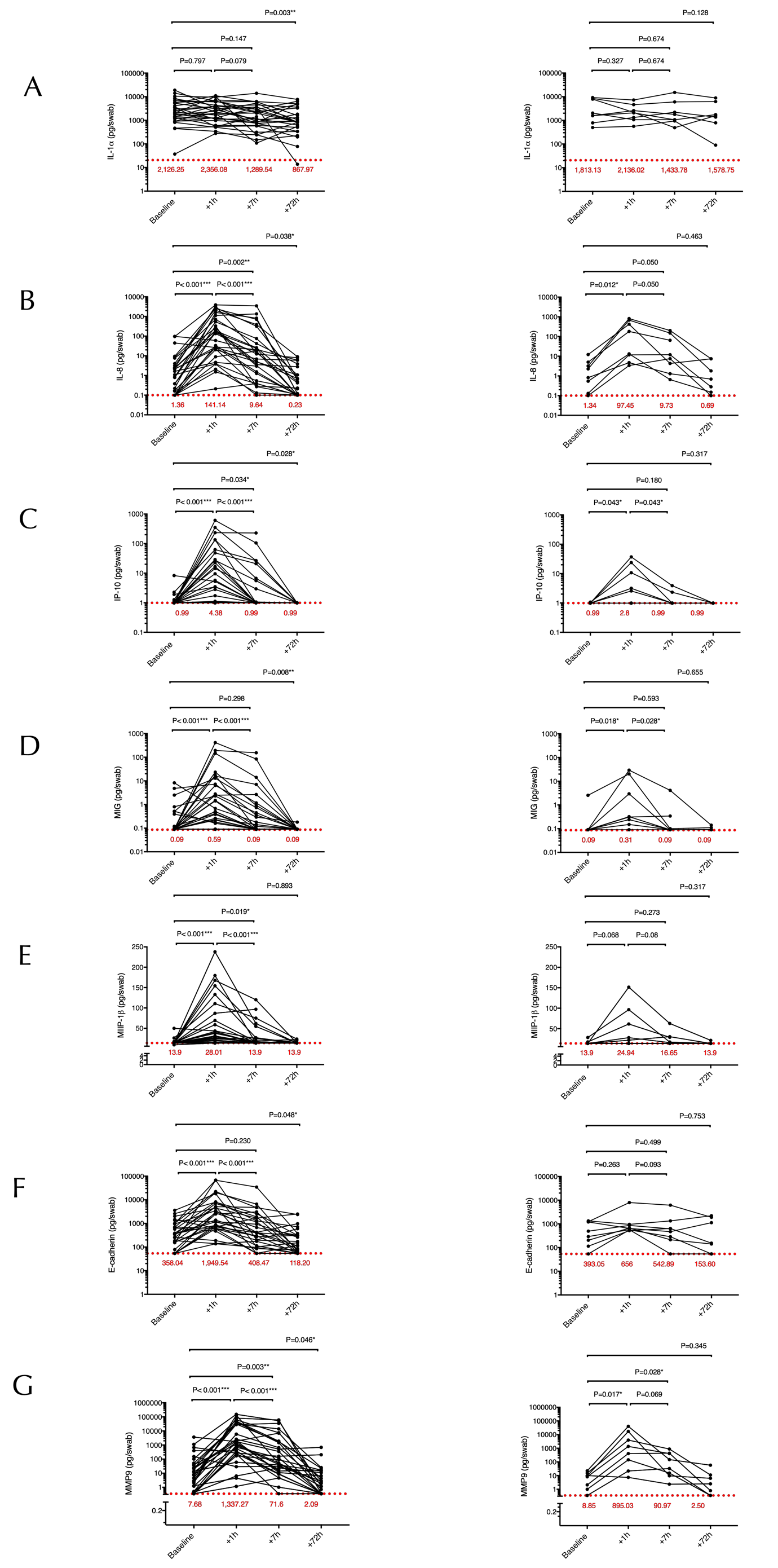
**

**S3 Fig. Impact of penile-vaginal sex on penile shaft immunology**. Time course of changes of **(A)** IL-1α (pg/swab) **(B)** IL-8 (pg/swab) (**C)** IP-10 (pg/swab) (**D)** MIG (pg/swab) **(E)** MIP-1β (pg/swab) **(F)** E-cadherin (pg/swab), and **(G)** MMP9 (pg/swab) after condomless sex (N=30) and condom-protected sex (N=8). The red dotted line represents immune parameter LLOD, and red numbers the median cytokine concentration at each visit. Statistical comparisons were performed using two-tailed Wilcoxon Signed Ranked test.
