# Supplementary material for: Insertive condom-protected and condomless vaginal sex both have a profound impact on the penile immune correlates of HIV susceptibility
Source: PLoS Pathog. 2022 Jan 4;18(1):e1009948. doi: 10.1371/journal.ppat.1009948 (PMC8769335; doi:10.1371/journal.ppat.1009948)
Supplement: S4 Fig — Coronal sulcus cytokine levels (pg/swab) in uncircumcised (N = 16) and circumcised (N = 14) men. The red dotted line represents immune parameter LLOD. Statistical comparisons were performed using two-tailed Mann-Witney U test. (DOCX) [file ppat.1009948.s004.docx]

**S4 Fig. Circumcision status and baseline penile immune parameters.**

**S4 Fig. Circumcision status and baseline penile immune parameters.** Coronal sulcus cytokine levels (pg/swab) in uncircumcised (N=16) and circumcised (N=14) men. The red dotted line represents immune parameter LLOD. Statistical comparisons were performed using two-tailed Mann-Witney U test.
