# Supplementary material for: Insertive condom-protected and condomless vaginal sex both have a profound impact on the penile immune correlates of HIV susceptibility
Source: PLoS Pathog. 2022 Jan 4;18(1):e1009948. doi: 10.1371/journal.ppat.1009948 (PMC8769335; doi:10.1371/journal.ppat.1009948)
Supplement: S5 Fig — Cells were gated on lymphocytes, singlets, live, CD3+ cells, CD4+ cells, CD4+T cells expressing β7 high. (DOCX) [file ppat.1009948.s005.docx]

**S5 Fig. Gating strategy and representative plots for CD4+β7 ^high^ T cells in blood.**

**
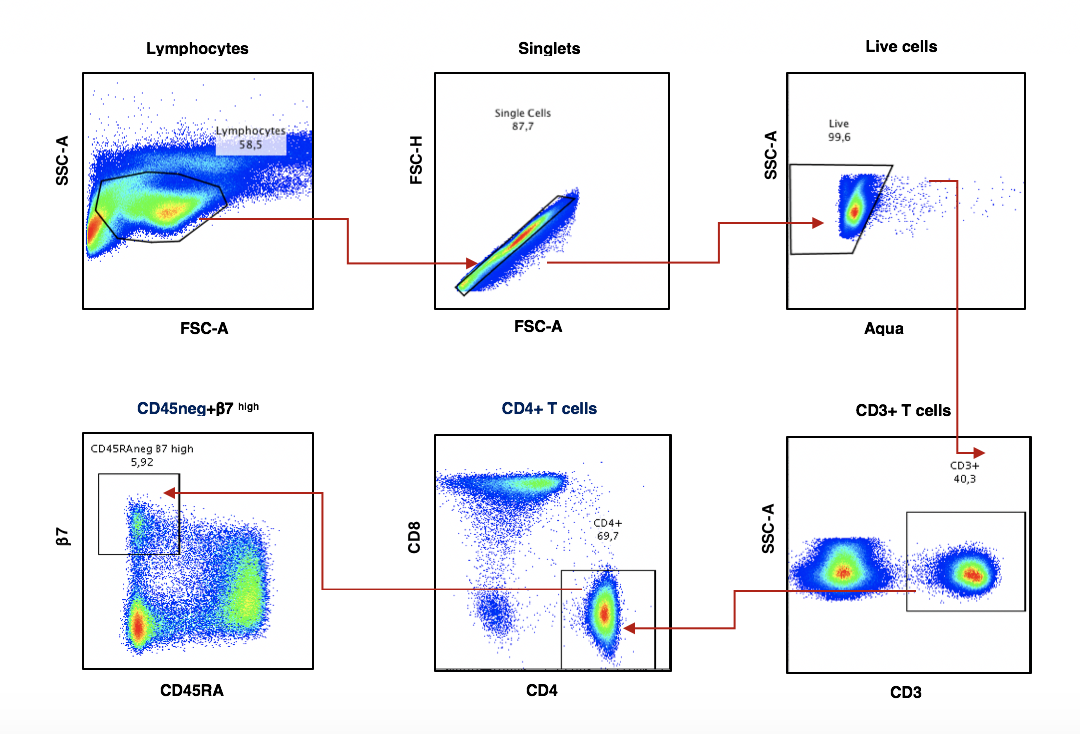
**

**S5 Fig.** **Gating strategy and representative plots for CD4+β7 ^high^ T cells in blood.** Cells were gated on lymphocytes, singlets, live, CD3+ cells, CD4+ cells, CD4+T cells expressing β7 ^high^.
