# Supplementary material for: Insertive condom-protected and condomless vaginal sex both have a profound impact on the penile immune correlates of HIV susceptibility
Source: PLoS Pathog. 2022 Jan 4;18(1):e1009948. doi: 10.1371/journal.ppat.1009948 (PMC8769335; doi:10.1371/journal.ppat.1009948)
Supplement: S1 File — (DOCX) [file ppat.1009948.s006.docx]

**Swab collection – Uncircumcised**

**Required items**

- Four (4) pre-labeled vials
- Four (4) swabs
- A larger container containing liquid (“normal saline”)
- A pair of gloves
- Bag for vials
- Urine cup
- Bag for urine cup

**Procedure:**

- Ensure genital area is well exposed
- Wear a pair of gloves
- Please do not touch other areas than the ones indicated in the instructions

**1 of 3: Shaft swab collection**

1.
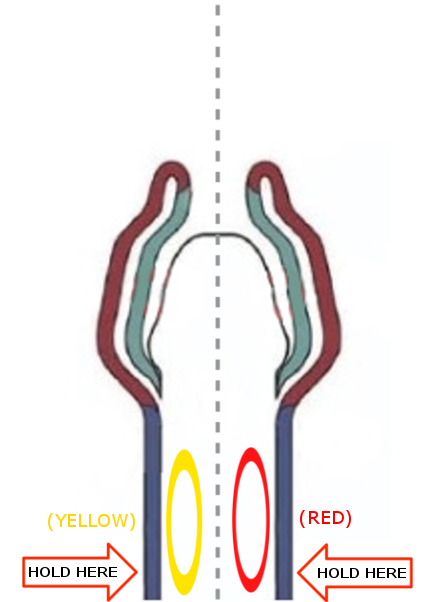
One swab sample is needed from **left side of shaft (yellow oval beside “YELLOW”)** and one from **right side of shaft (red oval beside “RED”)**.
2. Pick one swab and pre-moisten it in the liquid provided in the larger container labelled “normal saline.”
3. Remove excess liquid by squeezing the swab tip onto the inside of the container.
4. Using less dominant gloved hand, hold the shaft at the point shown by arrows.
5. Gently but firmly roll the pre-moistened swab on **left** side of shaft (yellow oval) 4-5 times.
6. Carefully place the swab in **vial with yellow cap**.
7. Carefully break the swab against the rim of the vial and put the cap on tightly, leaving swab tip inside.
8. Repeat steps 3 to 7 with a different swab except this time on the **right** side (red oval) then place the swabs in **vial with red cap**.
9. Carefully break the swab against the rim of the vial and put the cap on tightly, leaving the swab tip inside.

***Please turn page over.***

**2 of 3: Glans and sulcus swab collection**

1. ***
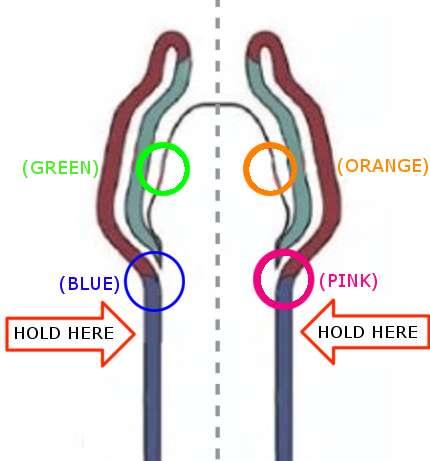
***One swab sample is needed from **left side of glans (green circle beside “GREEN”)** and **left side of sulcus (blue circle beside “BLUE”)**. The second swab will be used on the **right side of glans (orange circle beside “ORANGE”)** and the **right side of sulcus (pink circle beside “PINK”)**.
2. Pick one swab and pre-moisten it in the liquid provided in the larger container labelled “normal saline.”
3. Remove excess liquid by squeezing the swab tip onto the inside of the container.
4. Using less dominant gloved hand, hold head of penis at the point shown by arrows.
5. Gently retract foreskin to expose head of penis.
6. Gently but firmly roll the pre-moistened swab on **left** side of glans (head of penis **under the foreskin** – green circle) 4-5 times.
7. Gently retract foreskin to expose sulcus area (groove between head and shaft of penis).
8. Gently but firmly roll **the same** pre-moistened swab on **left** sulcus area (blue circle) 4-5 times.
9. Carefully place the swab in **vial with green cap**.
10. Carefully break the swab against the rim of the vial and put the cap on tightly, leaving swab tip inside.
11. Repeat steps 2 to 10 with a different swab except this time on the **right** side (orange circle and then pink circle) and place the swab in **vial with orange cap**.
12. Remove your gloves then put all the vials and the larger container back in the plastic bag and seal it.
