# Supplementary material for: Insertive condom-protected and condomless vaginal sex both have a profound impact on the penile immune correlates of HIV susceptibility
Source: PLoS Pathog. 2022 Jan 4;18(1):e1009948. doi: 10.1371/journal.ppat.1009948 (PMC8769335; doi:10.1371/journal.ppat.1009948)
Supplement: S1 Table — (DOCX) [file ppat.1009948.s008.docx]

**S1 Table. Comparison of baseline characteristics between condomless and condom-protected group.**

| Characteristic | Condomless Condom | p-value |
| --- | --- | --- |
| **Age**  **(Median, range)** | 22 23  (18-44) (19-40) | p>0.99 |
| **Relationship length**  **(Median, range)** | 18.5 18  (1-96) (3-100) | 0.847 |
| **Circumcised % (n)** | 46.7 (14) 62.5 (5) | 0.693 |
| **STIs history % (n)** | 20 (6) 0 (0) | 0.309 |
| **BV (female partner) % (n)** | 16.7 (5) 0 (0) | 0.563 |
| **Penile washing % (n)** | 63.3 (19) 62.5 (5) | p>0.99 |

***** One couple participated twice, once in the condomless and once in the condom-protected group, with a four-months interval between them.
