# Supplementary material for: Insertive condom-protected and condomless vaginal sex both have a profound impact on the penile immune correlates of HIV susceptibility
Source: PLoS Pathog. 2022 Jan 4;18(1):e1009948. doi: 10.1371/journal.ppat.1009948 (PMC8769335; doi:10.1371/journal.ppat.1009948)
Supplement: S2 Table — (DOCX) [file ppat.1009948.s009.docx]

**S2 Table. Proportion of cytokines detectable in penile swabs at baseline.**

| Cytokines | Coronal Sulcus  (% detectable) | Shaft  (% detectable) |
| --- | --- | --- |
| **IL-1α** | 100% | 100% |
| **IL-8** | 97.4% | 81.6% |
| **E-cadherin** | 92.1% | 84.2% |
| **MMP-9** | 92.1% | 84.2% |
| **MIG** | 57.9% | 26.3% |
| **IP-10** | 36.8% | 18.4% |
| **MIP-1β** | 21% | 21.6% |
| **MIP-3α** | 7.9% | 15.8% |
| **IL-6** | 5.3% | 7.9% |
| **IL-17** | 5.3% | 5.3% |
| **IFN-α2a** | 5.3% | 5.3% |
