# Supplementary material for: Insertive condom-protected and condomless vaginal sex both have a profound impact on the penile immune correlates of HIV susceptibility
Source: PLoS Pathog. 2022 Jan 4;18(1):e1009948. doi: 10.1371/journal.ppat.1009948 (PMC8769335; doi:10.1371/journal.ppat.1009948)
Supplement: Attachment — Submitted filename: AM_reviewers response.docx [file ppat.1009948.s010.docx]

**Response to reviewers:**

Thank you very much for submitting your manuscript "Insertive vaginal sex has a profound impact on the penile immune correlates of HIV susceptibility" for consideration at PLOS Pathogens. As with all papers reviewed by the journal, your manuscript was reviewed by members of the editorial board and by several independent reviewers. The reviewers appreciated the attention to an important topic. Based on the reviews, we are likely to accept this manuscript for publication, providing that you modify the manuscript according to the review recommendations.

*We are delighted to see that the reviewers found the study topic important, and have revised the manuscript thoroughly based on their comments. All the changes are highlighted in yellow in the appended revised manuscript.*

Please consider comments from reviewer #2 including revising the title e.g. "Insertive condom and condomless vaginal sex increases penile immune correlates of HIV susceptibility" (or similar) and analysing the data in a different way to see if elevated penile IL-8 and MIG levels following sex remain significant. Please address the cell analysis and the results of other markers used and include in the supplementary section and discussion. Some of the figures are too small e.g. Fig 2 and need to be enlarged. Gating strategy (Fig 4) can be moved to the supplementary section. Please consider comments from reviewers aimed to increase clarity of the manuscript.

*We have adjusted the title based on the editor’s suggestion to “Insertive condom-protected and condomless vaginal sex both have a profound impact on the penile immune correlates of HIV susceptibility” We hope the change in the title is acceptable for reviewer #2.*

*As requested by reviewer #2, we also now represent cytokine changes using a heat map, and agree that this is an excellent addition. Fig 2 and 3 are now enlarged. The gating strategy is moved to the supplementary section as S5 Fig.*

I have also included additional minor revisions:

Page 5, lines 101- 103. Please provide citation to substantiate statement
Page 19, line 392. RPMI rather than RMPI?
Page 20 line 417. Provide xg force for centrifugation (i.e as done on line 420).

*Thank you, the required changes have been made.*

**Reviewer #1**

1. Fig 2 should be reworked to enlarge each graph so that the figure can more easily be interpreted. The layout of the figure is fine.

*We apologize for any lack of clarity. We have now enlarged each panel of Fig 2. While they are still relatively small (as will be the case in the eventual manuscript), the resolution of the figures is high, and so we invite the reviewer to also enlarge the figure on their screen, and resolution should remain clear.*

2. Fig 4A the flow cytometry gating strategy should be moved to supplementary data. Some of the data in the supplementary figures, such as S1 and S3 could be moved into main figures.

*We have moved Fig 4A to the supplementary data (now S5 Fig). Figure S1 focuses on the penile shaft, and we are not sure that this shaft-sulcus comparison adds enough to the paper to warrant inclusion in the main manuscript. Therefore for now we have left this as a supplementary figure, but can make the change if the reviewer feels strongly on this point*.

3. The inclusion of CD4+B7+ cell analysis is important as this subset has been shown to be susceptible to HIV infection. However, even though multiple other important markers were described in the methods, none of these results are shown or eluded too. These should be further discussed, even if no changes were observed and included in supplementary data.

*We apologize for this omission. The goal of our flow studies was particularly to assess the impact of sex on the frequency of B7-high CD4+ T cells in blood, since this has been linked to increased HIV susceptibility (hence our additional sub-analysis of changes based on circumcision status for this marker). However, we did indeed analyze multiple other T cell parameters, and the only overall change that we observed after condomless sex was a decrease in the proportion of regulatory T cells at 1 hour and 48 hours after sex. These results are now presented in the manuscript (lines 305-317).*

4. Although not crucial to the publication of this manuscript, performing microbial 16s analysis would also greatly improve the scientific rigor of this manuscript and could help explain the observed changes. The authors do mention that they are not powered to assess the impact of female BV on their observed results, but could discuss this in more detail as it is a very important facet of vaginal health.

*Thank you for this excellent suggestion. We have collected samples for microbiome analysis, but currently this work is not funded (two grant proposals have been submitted to obtain funding); nonetheless, we think that these very interesting immune changes merit publication in their own right.*

*The reviewer is correct that the diagnosis for BV by Nugent score is essentially a crude assessment of the FGT microbiome. Therefore, although with n=5 BV+ participants we are underpowered for the analysis, at the reviewer’s request we have now run this analysis. Since BV is linked to inflammation in the FGT and women with BV have high levels of IL-1α, we specifically compared the level of penile IL-1α and the magnitude of change in penile Il-1α after condomless sex between BV+ (Nugent score>7) versus BV- (Nugent score <7) female partners. We did not observe any significant differences between these two groups. This has now been clarified in the manuscript (lines 354-358).*

5. The authors need to include how BV was assessed in their methods section as this is missing.

*We apologize for this omission. This information is now included in the Methods section (lines 439-441)*.

**Reviewer #2**

1. It is unclear how the authors conclude cytokines are derived from the female cervico-vaginal tract when the levels of IL-8 and MIG show somewhat similar magnitudes and kinetics in men wearing condoms (Fig 2). This should be more carefully explained and currently is unclear.

*We apologize if we did not make this point clear in the original manuscript draft. Essentially, during penile-vaginal sex the coronal sulcus will be directly exposed to one of two biological fluids: either female genital secretions (during condomless sex) or pre-ejaculate and semen (during condom-protected sex). Since both vaginal secretions and semen are enriched for IL8 and MIG, we believe that this is the likely cause of the post-sex increase, and have now made this more clear in our Results and Discussion sections (see lines 261-264).*

2. Related to this, it is also unclear why cytokines in the FGT and semen are in a supplementary table, when it is a feature of the conclusions to the paper. This should be considered to placed in the main body of the MS.

*Thank you, this is a great suggestion. We have now moved the S2 Table into the main manuscript as Table 2.*

3. Results from lines 237-257 on penile changes reflect exposure to cytokine-rich fluids during sex is quite confusing. It is not clear whether the results are linked to a Table or a figure.

*We apologize if the wording of our analytic approach here is confusing. We do not have additional figures on which the analysis is based, but in the revised manuscript we have worked with our biostatistician to explain our approach more clearly (please see lines 259-281). Our approach involved performing ANOVA alone, and then including the baseline concentration of cytokines in CVS as a covariate in order to assess their impact on the statistical associations seen. Essentially this is a form of mediation analysis, and our results showed that the immune changes were likely induced by CVS cytokines rather than by the act of sex itself.*

*The first paragraph refers to Table S2, which shows cytokine levels in semen and CVS. We have now moved this table into the main manuscript as Table 2, and it is referenced in the first paragraph of this section (line 261).*

4. Lines 280-288 on homing integrin expression.
• Why did the authors look in blood – a clear rationale is missing.

*We apologize for any lack of clarity. This is related to the fact that mucosal “disruption” (either gut biopsy or penile circumcision) has been shown to alter T cell expression of homing receptors in blood [Lemos, PLoS One, 2016], and that the expression of homing receptors in the blood has been linked to increased HIV risk [Sivro A, Sci Trans Med, 2018]. This is now clarified in lines 306-310.*

• Why did the authors only show CD4+beta-7 when alpha-4 was used in the flow panel. The authors cite a very old reference from 1993 as some form of justification.

*The current convention is to show beta7-high staining as a proxy for a4b7 staining, and this was the approach used in the study linking blood expression of a4b7 with heterosexual HIV acquisition [Sivro A, Sci Trans Med, 2018]. Therefore, we felt that this was the most appropriate approach to use in this study.*

• Is there a reason why the authors used a gut homing marker as opposed to CLA – which would be more appropriate to know which cells in the systemic circulation are migrating to the skin.

*That is an interesting question. Our prior studies have demonstrated that CLA expression is high on CD4+ T cells derived from both the inner and outer foreskin [Galiwango R, AJRI 2019], but this integrin has not been linked to HIV acquisition while a4b7 has been, both in vitro and in vivo. In retrospect it would have been ideal to include both integrins, but unfortunately we did not include CLA.*

• Why did the authors not include CCR5 expression, when it was part of the panel (according to the methods section).

*We apologize for not including these data. CCR5 was indeed studied, and expression levels did not change after condomless sex. The results are now added to the manuscript (line 305-317).*

5. The authors may need to change the title of the MS to reflect the data in the paper, as opposed to conjecture, or previous data (albeit from the same group).

*We have now adjusted the title to reflect our results in a more granular way. Based on the editor’s suggestion the title is changed to “Insertive condom and condomless vaginal sex both have a profound impact on the penile immune correlates of HIV susceptibility” We hope the change in the title is acceptable to the reviewer.*

6. It would be worth analyzing the cytokines in a different way and looking at fold changes in an unsupervised clustering, PCA and heat-maps; showing how clusters of cytokines may separate out between variables. Do IL-8 and MIG stand up to this approach?

*This is an excellent suggestion. We now also present the fold-change in cytokine/chemokine concentrations as a heat-map (Fig2 K, which we think is an excellent addition. We did not observe any clustering of cytokines based on sex group or circumcision status (S2 Fig).*

**Reviewer #3**

Background.

1. Nicely written. Importance of IL-8 is described but not for MIG. Suggest to add a sentence or two in paragraph 2 of background, since this is a primary endpoint as stated in Methods.

*We thank the reviewer for their positive feedback. We have now added some discussion of MIG as well as IL8 to the Introduction section (lines 84-88).*

2. Hypotheses clearly stated and supported.

*Thank you.*

Methods.

1. Penile swabbing:

1a. Could authors please provide more detail on how the penile swabs were self-collected? E.g., were there demonstrations, pictorial guidance, protocol (amount of passes or time), etc. to ensure standardization?

1b. How much of the shaft was swabbed (e.g., were men directed to swab the whole of it, or maybe a centimeter or so?)

1c. Could the authors please clarify about samples being “collected left and right side of the penis”? The subsequent part of the sentence says “coronal sulcus/glans” and then “shaft”, so what parts of the penis were “collected from left and right”?

*The procedure was explained in detail by the research coordinator, based on an in-person review of a detailed instruction sheet that was then provided to participants for their reference. This is now attached as a supplementary file (S2 file), and we hope that this addition addresses the reviewer’s questions.*

d. What type of swabs were used? E.g., flocculated, pre-moistened, etc.

*Flocked swabs pre-moistened in normal saline were used for penile swab collection. This information has now been added to the Methods section (lines 423-426).*

2. I don’t recall seeing the Nugent scoring to identify BV (though it says a slide was smeared), but it’s in the footnote of Table 1, but should include it in Methods.

*We apologize for this oversight; this information is now included in the Methods section (lines 439-441).*

3. As there was such a smaller n of condom protected sex, how was participation in the “condomless sex group” vs. “condom-protected sex group”. Presumably, participants self-selected?

*While we prespecified that a relatively small subset of participants would be observed after condom-protected sex, couple self-selected as to whether or not a condom would be used. We have now added this to the Methods section (lines 125-126).*

Results

1. Table 1.

1a. Age – “18-44” is likely range. Please add (range) after Age in the Characteristic column. Same for other continuous variables.

*The ranges for all continuous variables are now included in Table 1.*

1b. Presumably continuous variables are presented as medians? Or maybe they’re means. Please specify in the table.

*Continuous variables are presented as medians. This is now specified in Table 1.*

1c. Why are some variables reported as % (e.g., self-reported STI, penile washing, female partner BV) while others are reported as n? Needs to be consistent throughout.

*The units and participant numbers are now added for STI history, penile washing, and BV status of the female partner.*

1d. How was penile washing defined? Was this primarily among uncircumcised men?

*In the questionnaire, we asked whether men wash their penis aside from regular bathing. So, it is based on self-declaration. The number of men practicing penile washing was similar comparing uncircumcised men to circumcised men (12 versus 11, respectively).*

2. Figure 1 is nice. Figure 2 and 3 graphs are useful, but very tiny for this reviewer; I had to go to 200%. Hopefully journal can represent that better in publication or maybe most other readers have larger monitors. Or better eyesight.

*Sorry about this - other reviewers have also noted that Fig 2 should be enlarged. We have now done so, and also enhanced figure resolution to permit enlargement.*

- The baseline data showing no differences by any covariates should be added to the Supplementary files.

*Thank you for your suggestion. A comparison of important baseline characteristics between condomless and condom-protected groups has now been added as S1 Table.*

3. Lines 230-231: “unexpected the immediate increase in coronal sulcus MIG levels tended to be more marked in circumcised men vs.” (presumably uncircumcised men).

*This typo has now been corrected.*

4. It’s a small sample size with multiple comparisons.

*There is no question that this was a study limitation, and one that is highlighted in the “study limitation” section of the discussion. We deliberately focused on predefined primary endpoints IL-8 and MIG based on their association with HIV acquisition in cohort studies, to reduce concerns around multiple comparisons – but many variations in biology and sexual behaviour may affect results, meaning that subsequent sub-analyses should be considered exploratory.*

Discussion

- Is it known how penile cytokines change/don’t change after masturbation? This would be an additionally interesting control.

*To the best of our knowledge, this has not been studied – but we agree that it would be an interesting area for future research.*

- If these changes occur regardless of condom use – by exposure to cervicovaginal secretions if condomless sex or by exposure to man’s own semen after condom protected use – could this also in part be a physiological response, such as after exercise which also induces IL-8? Authors posit that the increases “were therefore likely mediated via direct contact with these genital fluids”; but the cervicovaginal fluids vs. semen likely have different “things” in them eliciting response?

*This is an excellent point. The levels of certain immune factors (MMP9, etc) were quite different in semen and CVS, and was part of the reason why we performed the mediation analysis separately for condomless and condom protected sex. While that analysis showed that most of the change in penile surface cytokines was driven by cytokine levels in CVS/semen, we cannot rule out that more subtle changes may be driven by tissue responses to sex itself, and such endogenous changes would theoretically be more likely to have an impact within the penile tissues themselves. This possibility is now noted in the Discussion (lines 381-82, page 19).*

- Median duration of relationship was 18 months, with IQR 6.5 to 36 months. With so few having "newer" relationship, is it possible that the “impact” of sexual activity attenuates over time? Reviewer understands the sample size is small and that “new” relationship vs. more established, but could this perhaps contribute to lack of differences in some expected areas?

*This is excellent question. We originally hypothesized that couples who had been together for a shorter time would demonstrate greater post-sex immune changes, but overall, we saw no significant correlation between the prior duration of the relationship and the extent of penile cytokine changes after condomless sex. A sentence to this effect has now been added to the Results section (lines 221-223).*

- Lines 325-335 on why no difference observed by circumcision status: could the authors please add some details to “future studies” "would be interesting" so that the sentence has more meaning/contribution?

*While studies to define the immune correlates of HIV in circumcised men would indeed be interesting, this is probably not the best place to explore a topic that is at best tangential to the focus of the current manuscript. Therefore, we have opted to remove this part of the discussion, and hope that the review agrees this is appropriate.*

- What might be happening with cytokine/chemokine response for those who have penile-vaginal sex ongoing/several times per week? Would that provide different explanatory information with regards to HIV risk?

*The current protocol was designed to ensure periods of abstinence prior to baseline sampling, prior to sex for the purpose of the study and during the follow up sampling period. While not studied, based on our results we would hypothesize that very frequent sex would give a more continuous high level of chemokines on the penile surface, and perhaps lead to more epithelial disruption. However, since this is conjecture we have not included these ideas in the manuscript.*
